# Personalized Medicine in Allergic Asthma: At the Crossroads of Allergen Immunotherapy and “Biologicals”

**DOI:** 10.3389/fped.2017.00031

**Published:** 2017-02-17

**Authors:** Benedikt Fritzsching

**Affiliations:** ^1^Pediatric Pulmonology and Allergy, Children’s Doctor Service, Heidelberg, Germany; ^2^Heidelberg University Hospital, Heidelberg, Germany

**Keywords:** allergy, asthma, personalized medicine, stratified medicine, allergen immunotherapy, biologicals, monoclonal antibodies

## Abstract

Major allergic disease can be viewed as clinical syndromes rather than discrete disease entities. Emerging evidence indicates that allergic asthma includes several disease phenotypes. Immunological deviation toward high T helper cell type 2 cytokine levels has been demonstrated for a subgroup of pediatric asthma patients, and now, several novel monoclonal antibodies have been approved for treatment of this subgroup as a stratified approach of “personalized” medicine in allergy. Introduction of component-based IgE testing before allergen immunotherapy (AIT), i.e., testing for IgE cross-reactivity before initiation of AIT, has also brought stratified medicine into allergy therapy. Improved responder criteria, which identify treatment-responders previous to therapy, might foster this stratification and even individualized AIT might have an impact for tailor-made therapy in the future. Furthermore, combining antibody-based treatment with AIT could help to establish more rapid AIT protocols even for allergens with a high risk of anaphylactic reactions. Efforts to advance such “personalized” medicine in pediatric allergy might be challenged by several issues including high costs for the health-care system, increasing complexity of allergy therapy, the need for physician allergy expertise, and furthermore ethical considerations and data safety issues.

## Introduction

Over recent emerging evidence has shown that major allergic diseases represent syndromes rather than single disease entities (1, 2). Specifically, pediatric asthma reflects several phenotypes, which might reflect different mechanisms called “endotypes” (2). As a major endotype, immunological deviation toward high T helper cell type 2 (Th2) cytokine levels in a subpopulation of asthma patients has been proposed by several studies. Novel monoclonal antibodies have been demonstrated to ameliorate disease in “Th2high” patients and other subpopulations. Here, we focus on antibodies interfering with Th2 cytokines (IL-4, IL-5, IL-13). A number of other antibodies such as anti-thymic stromal lymphopoetin have been tested but are not currently available to patients including children. Beyond clinical characteristics, studies on biomarkers are being developed to identify patient subpopulations who might benefit from novel therapy in contrast to non-responders (3). Such stratification strategies coined the term “stratified medicine” or “precision medicine” in allergy treatment. In allergy, truly individual treatment, i.e., individual composition of recombinant allergens for allergen immunotherapy (AIT) is not available and limited by regulatory processes in many countries (4, 5). Of note, “personalized medicine” is considered more than “treating the right patient with the right drug at the right time” since every patient–doctor relationship includes personal aspects of disease. Here, “stratified medicine” will be used as the more precise term instead of “personalized medicine.”

## “Th2high” Asthma

Development and market authorization of “biologicals” termed monoclonal antibodies is an demanding process for the manufacturer and also approved drugs like Omalizumab are still of limited use in the field. Whereas recruitment of study patients to fill subpopulation groups is challenging in study planning, approved antibodies are high priced and limit their broad acceptance and usage in the field. Given small patient subpopulations in clinical trials, the risk of failure in follow-up studies is higher in stratified medicine. For example, airway epithelium-derived protein periostin was helpful to identify asthma patients with high expression levels of the Th2 cytokine IL-13 (3) and application of the anti-IL-13 maAb Lebrikizumab ameliorated asthma in these patients (6). Of note, application of Lebrikizumab to the whole asthma patient group showed little or no effect (6). Intriguingly, follow-up studies [LAVOLTA I and II (7)] also revealed conflicting data for the treatment with Lebrikizumab in the “Th2high” patient subgroup, and therefore, Lebrikizumab will not be followed up as an asthma drug by the manufacturer. Nevertheless, these efforts demonstrated that in allergic asthma patient subpopulations exist, who might benefit from a specific treatment whereas other patients might not. Earlier in 2016, anti-IL-5 antibodies Bendralizumab, Mepolizumab, and Reslizumab were FDA approved for the treatment of the subgroup of patients with severe esoinophilic asthma. It is noteworthy, that when such studies were performed almost 15 years ago in the broader asthma population, few convincing effects were observed. With the emerging concept of stratified medicine in allergy, follow-up studies highlighted the importance of defining subpopulations to demonstrate significant effects in not all but few asthma patients defined by several criteria. With a focus on patients with “Th2 high” allergic asthma several biomarkers have been proposed. Specificity of such “Th2 high” surrogate markers remains controversial, i.e., increased blood eosinophil levels were used to define patients who might benefit from treatment with Dupliumab, an IL-4 receptor α-blocking antibody. However, also patients with lower blood eosinophil counts showed significant improvement of asthma (8). Obviously, current definitions of “Th2 high” asthma are difficult to use in the clinical setting. This may not just be due to insufficient surrogate markers for definition of “Th2 high” asthma but also due to significant variability of this endotype with discrete subtypes among patients with asthma.

## From Bench to Bed and Back: “Biologicals” in Allergy

Bringing these so-called “biologicals” into the market of major public health system is not only demanding for the manufacturer. Costs for novel antibody-based therapies frequently exceed several tens of thousands of Euro/US dollar per year for a single patient. Furthermore, defining the target population is still a dynamic process, since most antibodies interfere with key molecules (i.e., IgE, IL-4/IL-13, IL-5) in different major allergic diseases. Lebrikizumab was considered to be used for a subgroup of “Th2high” asthma patients. In fact, this will not be followed up. Instead, testing for atopic dermatitis comes into play. Similarly, Dupliumab shows high potential for atopic dermatitis therapy (9) even before being approved for asthma treatment. Omalizumab has previously been approved for treatment of chronic urticaria (10)—years after market authorization for severe asthma patients. Collectively, identification of patients with highest benefit for a given drug is a dynamic process even in the age of companion diagnostic tests with biomarkers used to define subgroups.

For research, data from clinical studies with antibodies are of major importance. Translation of knowledge from small animal models is very limited and frequently used genetic knockdown approaches cannot easily be followed up in humans. With the application of highly specific antibodies, this translational “gap” is getting smaller. Furthermore, clinical responses to antibodies might help defining asthma phenotypes much better than before, and this asks for better design of animal models based on lessons learnt in the patient.

## AIT: Responder Versus Non-Responder

Treating allergies by application of proteins has been successfully performed by AIT for several years. It is well known that some patients respond to AIT very well, whereas other patients show almost no effect above substantial placebo effects (11). In general, it is not just subcutaneous immunotherapy (SCIT) that has been proven as efficient, but also sublingual immunotherapy (SLIT) (12, 13). However, only a few high-quality studies have been published so far for children, and more studies are necessary to extend the number of evidence-based AIT preparations for SCIT or SLIT in pediatric AIT. Considered especially important are studies on the prevention of allergic asthma. Finally, data derived from study cohorts need to be translated into “real-life.” Introduction of component-based IgE testing has helped to identify patients with higher risk to fail or benefit in AIT, especially in patients with multiple allergic sensitizations, i.e., grass and birch pollen allergies (14). Preliminary data suggest that higher levels of allergen-specific IgE could be helpful to predict AIT efficacy, i.e., IgE levels to house dust mite in children (15). Similar progress has been made in venom immunotherapy (VIT) for patients with anaphylaxis due to Hymenoptera stings. Differentiation between true sensitization and cross-reactivity based on cross-reactive carbohydrate determinants free species-specific allergens improved the selection of the appropriate VIT (16). Furthermore, patients with honey bee venom allergy and predominant Api m 10 sensitization have an increased risk of treatment failure due to a lack of Api m 10 in many preparations (17). In future, prospective studies are required to confirm these results derived from retrospective studies.

Further work is required to give the physician substantial diagnostic tests that can applied with ease to separate AIT responders from non-responders. Unbiased screening of biomaterial collected from clinical trials previous to approval by national regulatory authorities might help identification of novel biomarkers for detection of AIT responders beyond specific IgE antibodies. From a therapeutical point of view, much progress has been achieved in standardization of AIT products, mostly due to regulatory processes, i.e., approval by institutions like the Paul-Ehrlich Institute in Germany and novel regulations like the German Therapieallergene-Verordnung (18). However, regulatory processes have constantly to be followed up. Market authorization of individual AIT preparations defined by definition of individual allergic sensitization profiles, i.e., based on component-based IgE technology, is currently limited by regulation (19). Similar to many blood-derived good manufacturing practice products, approval of the production process might replace individual product approval in the future.

Following market authorization, acceptance in the allergy field is required to establish stratified or even individualized medicine as an integral part of the daily working routine in allergy treatment. Expert knowledge needs to be acquired from the physician and continuous communication from doctors with their patients but also with regulatory institutions, and many other key players in health-care systems are warranted to allow sustained progress. Since many therapeutical innovations focus on allergic asthma in children, there is a need for more pediatricians with expert training in allergy and pulmonology. Of note, risk factors of stratified or individualized medicine like data safety issues and increasing costs ask for participation of the general population into the discussion about stratified medicine in a given health-care system. Regulatory institutions need to keep up with novel treatment options and diagnostic tests in allergy.

## Co-Administration of AIT and “Biologicals”

In the future, application of biologicals together with AIT might improve and extend AIT. For example, MacGinnitie and colleagues reported early in 2016 that application of Omalizumab in patients with peanut allergy allowed rapid oral desensitization with high doses of peanut allergen (20). It is known, that intralymphatic application of allergen allows much higher antigen doses in the lymph node than conventional biodistribution of allergen following subcutaneous (s.c.) injection (21). Furthermore, only few intralymphatic injections show similar or even faster efficacy than s.c. injection (22). This suggests that increasing allergen doses by conventional routes (s.c., per oral) might still have some potential for AIT optimization. Shorter protocols with higher doses of allergen might increase patient adherence to AIT but requires additional medication like Omalizumab to give protection from strong anaphylactic reactions. These efforts seem reasonable especially when novel predictive tests of AIT responders will allow identification of the right patient.

## Summary

Several novel drugs have been approved for “stratified” medicine in allergy and many more might follow. Treatment decision-making will get more complex, especially if responder criteria for a suggested therapy are weak. As “personalized medicine” comes of age, several challenges need to be considered including high costs for health-care systems, more complex study designs due to difficult patient recruitment for smaller subgroups, and data safety considerations in the age of pharmacogenetics. Furthermore, the increasing complexity of allergy therapy asks for physicians with expertise in allergy. Treating the right patient with the right drug at the right time continues to be a therapeutical challenge.

## Author Contributions

BF concepted, wrote, and revised the manuscript.

## Conflict of Interest Statement

BF served at Speakers’ Bureaus of Stallergenes and the advisory boards of ALK-Abelló and Novartis.
